# *Ewingella allii* sp. nov. isolated from a diseased onion plant in the Columbia Basin of Washington State, USA

**DOI:** 10.1007/s10482-025-02116-6

**Published:** 2025-07-16

**Authors:** Fanele Cabangile Mnguni, Gi Yoon Shin, Lindsey J. du Toit, Michael L. Derie, Teresa A. Coutinho

**Affiliations:** 1 Department of Biochemistry, Genetics and Microbiology, Centre for Microbial Ecology and Genomics/Forestry and Agricultural Biotechnology Institute, University of Pretoria, Pretoria, 0002 South Africa; 2 Department of Plant Pathology, University of California, Davis, CA USA; 3 Department of Plant Pathology, Washington State University, Mount Vernon, WA USA

**Keywords:** Bacterial onion bulb rot, Novel species, Bacterial plant pathogen

## Abstract

Isolation of strain 20WA0182^T^ from a diseased onion plant grown in the Columbia Basin of Washington State, USA, led to preliminary identification as a member of the genus *Ewingella*. The strain was characterised as a Gram-stain-negative, facultative anaerobe that is rod-shaped, motile with polar flagella, catalase positive, and oxidase negative. The strain 20WA0182^T^ isolated was pathogenic to yellow onion bulbs, weakly pathogenic on onion leaves of the cv. Ranchero, and caused a pathogenic response using the red onion bulb scale necrosis assay. Phylogenetic analyses using the 16S rRNA gene and four housekeeping genes, *atpD, gyrB, infB,* and *rpoB,* showed that strain 20WA0182^T^ formed a branch that clustered with *E. americana* strains, but on a separate node, indicating it is a novel species of this genus. Whole-genome sequencing of strain 20WA0182^T^ revealed a genome size of 4,604,541 nt, with 25 contigs and a G + C content of 53.8%, strain 20WA0182^T^ was 99.2% complete. The average nucleotide identity of strain 20WA0182^T^ compared with *E*. *americana* strains scores ranged from 92.85 to 93.96%, below the 95% threshold to classify strains as the same species. Similarly, dDDH scores were 56.0 to 56.2%, less than the 70% threshold required to delineate prokaryotes as the same species. Strain 20WA0182^T^ and *Ewingella* sp. CoE-038-23 shared the ANI score above 97.59% and 81.0% dDDH score to be classified as a novel species of *Ewingella*. As the type strain 20WA0182^T^ (= BD 3290^ T^ = LMG 33618^ T^) was pathogenic to onion bulbs and leaves, the name *Ewingella allii* is proposed. GenBank accession number = JAWUDN000000000.

**Supplementary Information:**

The online version contains supplementary material available at 10.1007/s10482-025-02116-6.

## Introduction

The genus *Ewingella* consists of bacteria that are Gram-negative, rod-shaped, facultative anaerobes that are catalase-positive and oxidase-negative (Grimont et al. 1983). Grimont et al. (1983) provided the first description of the genus and species *Ewingella americana* in 1983. The genus belongs to the family *Yersiniaceae*, under the order *Enterobacterales,* class *Gammaproteobacteria* and phylum *Pseudomonadota* (Grimont et al. 1983; Adeolu et al. 2016). *Ewingella* was previously a member of *Enterobacteriaceae*, together with other common onion bacterial bulb rot pathogens, including species of *Pantoea, Enterobacter* (Grimont et al. 1983; Gitaitis and Gay 1997; Schroeder and Toit 2010; Schwartz and Krishna 2016; Paudel et al. 2024). When *Enterobacteriaceae* was revised, *Ewingella* was classified as being part of *Yersiniaceae* family (Adeolu et al. 2016). The first described strains were all obtained from clinical samples deposited at the Center for Disease Control and Prevention in the United States, but it was unclear at that time how these strains impacted human hosts. Further investigations published in 1987 described *E. americana* as an opportunistic, nosocomial pathogen, as the species was isolated from clinical environments (Grimont et al. 1983; McNeil et al. 1987). Since then, this *Ewingella* strain has demonstrated ubiquity by the ability to thrive in diverse environments, including in the stomachs of molluscs, in water, and as a pathogen of mushroom stalks (*Agaricus bisporus* and *Flammulina filiformis*) (Müller et al. 1995; Chowdhury et al. 2007).

To date, one complete and 14 draft genomes of *E. ewingella* strains (Grimont et al. 1983) were available on the National Center for Biotechnology Information (NCBI) database at https://www.ncbi.nlm.nih.gov/genome/?term=ewingella. A study by Liu et al. (2020) comparing the genomes of *E. americana* strains isolated from different hosts highlighted that host origin does not impact the phylogeny of this species. However, mobile genetic elements were identified as influencing the evolution and adaptability of *E. americana* strains to survive in varied environmental conditions. Although limited literature exists on interactions between *Ewingella* and plants, this study provides an initial association point for a second species of *Ewingella*, identified from a diseased onion plant showing wet, necrotic lesions. Strains of *E. americana* have been found with other bacteria that occur commonly on the surface of onion plants and in onion bulbs, as well as with bacterial pathogens of onions mentioned (Starr and Burkholder 1942; Goszczynska et al. 2006; Schroeder et al. 2009; Coutinho and Venter 2009; Zaid et al. 2012).

**Fig. 1:**
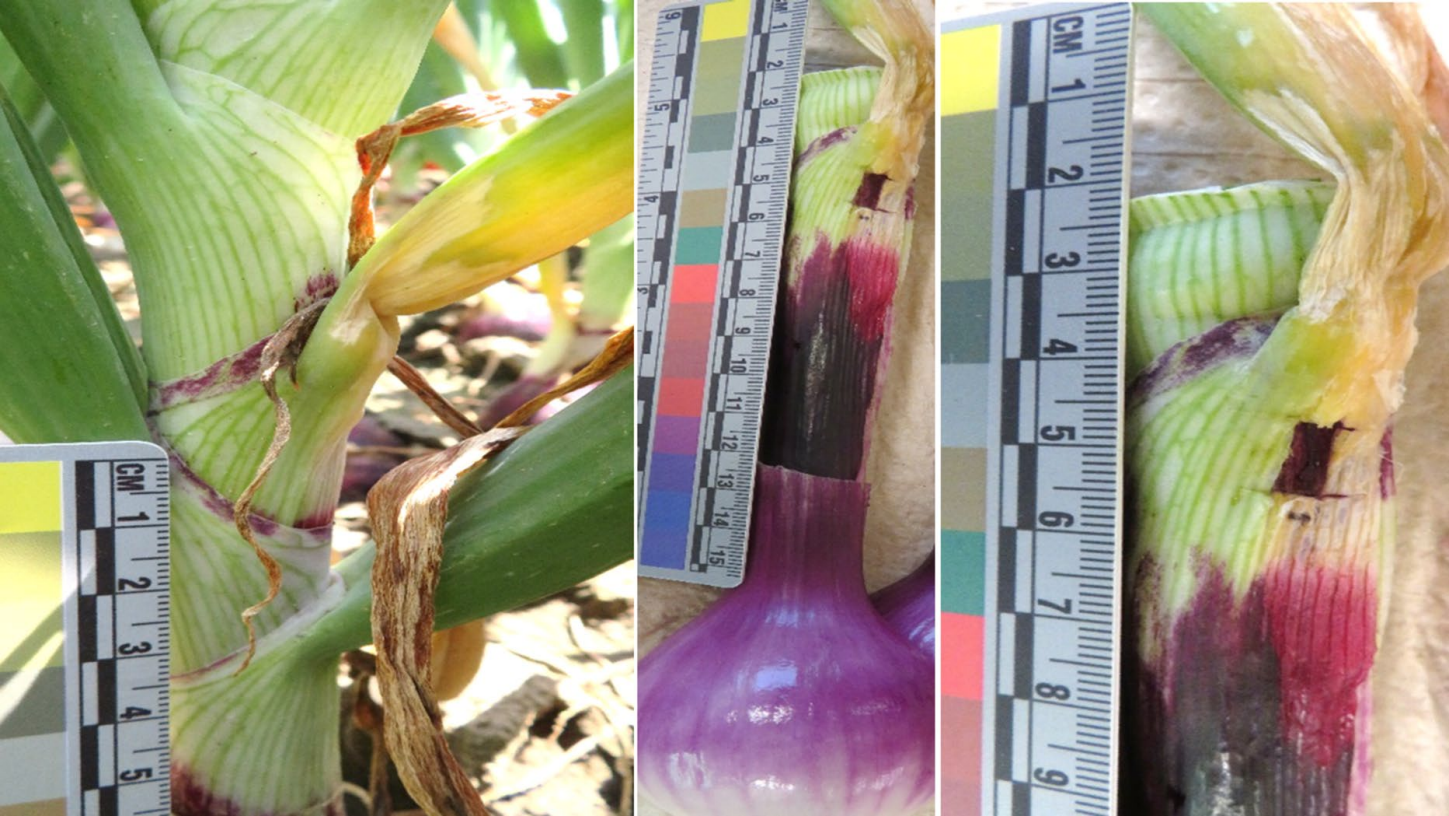
Symptoms of the original onion plant sampled from an onion crop in Washington State, USA on 30 July 2024 (left photo), from which strain 20WA0182^T^ of *Ewingella* was isolated. When the plant was cut lengthwise through the neck and bulb, and the outermost leaves and scales removed in the laboratory, a wet, necrotic lesion was observed extending from the point of attachment of the fourth-oldest leaf into the neck (middle photo). Isolation was carried out using a section of tissue sampled at the leading edge of the lesion, approximately 10 cm above the bulb (middle and right photos)

Onion production in the USA is valued annually at an estimated USD 1 billion, with Washington being one of the major onion-producing states where approximately 10,000 ha are planted for onion crops annually (USDA 2021, 2024). Bacterial pathogens can significantly contribute to the decrease in the yield of onion crops, leading to economic losses (FAO 2021; USDA 2023; NAMC 2023). The susceptibility of onion crops to bacterial pathogens is influenced by factors such as temperature conditions that favour bacterial growth in the field, postharvest and in storage (Belo et al. 2023a, 2023b). In this study, a novel species of *Ewingella* was isolated from a diseased onion plant sampled midsummer (on 30 July 2020) from a storage onion crop grown under centre pivot irrigation in the Columbia Basin of central Washington State. The plant displayed symptoms of bacterial rot at the point of attachment of the fourth-oldest leaf to the neck, with the lesion extending down the neck to ~10 cm above the bulb (Fig. 1). The sample was part of a three-year, multi-state survey of onion bacterial diseases in the United States, for Project No. 2019-1181-30013 funded by the Specialty Crops Research Initiative of the USDA National Institute for Food and Agriculture (https://alliumnet.com/stop-the-rot/). The bacterium was designated strain 20WA0182^T^ and identified to genus based on morphological, physiological, genotypic, and phylogenomic assessment.

## Materials and methods

### Isolation

Strain 20WA0182^T^ was isolated by crushing a ~5 mm^3^ piece of tissue taken from the leading edge of the symptomatic neck tissue in a drop of sterilized, deionized water. The macerated liquid was streaked onto the surface of the nutrient agar (NA) plate and incubated at 28 °C in the dark for 48 h (Schroeder et al. 2009; Schaad et al. 2001). Distinct and discrete colonies of strain 20WA0182^T^ were initially streaked onto NA plates three times to obtain a pure culture. Purified culture of strain 20WA0182^T^ was stored in 15% glycerol (v/v) at − 80 °C. Strain 20WA0182^T^ was classified as belonging to *Yersiniaceae* based on 16S rRNA gene sequencing. Sub-cultures of strain 20WA0182^T^ had been deposited in the Belgian Coordinated Collections of Microorganisms/Laboratorium voor Microbiologie Gent (BCCM/LMG) as strain LMG 33618^T^ and in the South African Agricultural Research Council (ARC) culture collection as strain BD 3290^T^.

### Pathogenicity tests

The bulb, foliar, and red scale necrosis (RSN) assays for pathogenicity were completed in the Vegetable Seed Pathology Program at the Washington State University (WSU) Mount Vernon Northwestern Washington Research & Extension Center in Mount Vernon, WA, United States, where strain 20WA0182^T^ originally was isolated. To compare our results, strain 20WA0182^T^ and the type strain of *E. americana*, CCUG 14506^T^ were tested for pathogenicity at the Centre for Microbial Ecology and Genomics at the University of Pretoria, South Africa.

Onion pathogenicity tests for strain 20WA0182^T^ and the type strain of *E. americana*, CCUG 14506^T^, were conducted at two different universities as mentioned above to evaluate strains pathogenicity towards onion bulbs, detached fleshy red onion scales, and onion foliage cv. Ranchero. *Pantoea ananatis* strain BD 251 known to be a pathogen of onion,was used as a positive treatment in our study (Stice et al. 2021), while 1X Phosphate-buffered saline (PBS made of 8 g NaCl, 0.2 g KCl, 1.44 g Na2HPO_4_, and 0.245 g KH_2_PO_4_ with pH 7.4) was used as a negative treatment for each pathogenicity test. The bacterial strains were each incubated in nutrient broth (NB) (Scharlab, Barcelona, Spain) on a shaker operated at 170 rpm for 24 h. The optical density (OD) of each suspension was measured at 600 nm (OD_600_), and the final concentration was adjusted to approximately 10^8^ CFU/ml. The inoculum of each strain was then centrifuged at 10 000 ×*g* (5424 R centrifuge, Eppendorf, Germany) for 2 mins. The supernatant was discarded, the pellet was resuspended in 1X PBS, and the suspension vortexed again for 2 mins.

In WSU , inoculum of strain 20WA0182^T^ was prepared overnight in nutrient yeast extract broth (NBY) broth on a shaking shaker at 150 rpm and 28 °C. Strains *P. ananatis* PNA97-1R and/or *Burkholderia gladioli* Bgd015 were used as positive treatments, and *Escherichia coli* Eco003 and sterile 1X PBS were included as negative treatments. A 0.5 ml aliquot of each bacterial suspension was pipetted into a sterile microcentrifuge tube, and the bacterial cells pelleted using a microcentrifuge operated at 14,800 rpm for 1 min. The supernatant was then removed, the pellet resuspended in sterile 1X PBS, and the OD_600_ measured and adjusted to 0.3 (~10^8^ cfu/ml) for use in the pathogenicity assays.

#### Bulb pathogenicity assay

University of Pretoria, the outer, dry, wrapper scales from store-bought yellow onion bulbs were removed. Strain 20WA0182^T^ and *E. americana* CCUG 14506^T^ were tested using 5 replicates of onion bulbs. Each bulb was injected with 0.5 ml of inoculum using a 21-gauge, 3.81-cm long needle inserted into the shoulder of the bulb. Sterile 1X PBS was injected into each of the 5 bulbs as a negative control treatment. The onion bulbs were incubated for 12-14 days at 28 °C then cut vertically through the point of inoculation. The cut surface area of each bulb was evaluated visually for symptoms of bulb rot indicated by the discolouration of inner fleshy scales and/or necrosis of the onion scales and asymptomatic as having no response to the strain inoculum.

For the bulb assay completed at WSU, a similar protocol was followed using yellow bulbs from a commercial field in central Washington. Once the outer dry scales were removed, the surface of each bulb was wiped with a paper towel moistened with 70% isopropanol. Five bulbs were used for each bacterial strain or treatment. A permanent marker was used to circle an area on the upper shoulder of each bulb to mark the inoculation site. The bulbs were each inoculated by puncturing the bulb at approximately a 45 angle through the marked area towards the center of the bulb and injecting 0.5 ml of inoculum as described above. The bulbs were incubated in the dark at 30 °C for 14 days, cut longitudinally, and evaluated for symptoms as described above.

#### Red scale necrosis assay

RSN assay was conducted following the method described by Stice et al. (2018). Briefly, the dry outer scales from the red onion bulbs were removed on and were cut into 3 cm x 3 cm sections using a sterile scalpel. For each strain of 20WA0182^T^, CCUG 14506^T^, and BD 251, three replicates of red onion fleshy scale pieces each were punctured in the centre on the top of the convex (outer) surface using a sterile 10 µl-pipette tip (Lasec,Pretoria, South Africa), and a 10 µl drop of the bacterial suspension placed carefully onto the punctured wound. Three red onion scale pieces were treated with 1X PBS as a negative treatment. The scales were then placed in a 184mm (L) x 184mm (W) x 152mm (H) plastic container (Addis, Pretoria, South Africa) on top of paper towels that had been dampened with double-distilled water to create a humid chamber. The scales were incubated for 4 days at 28 °C. Then each scale was evaluated visually for evidence of necrosis based on the clearing of the red pigment around the inoculated wound site, as described by Stice et al. (2018). The positive RSN test was determined by the clearing of the red pigment of at least 2 of the 3 of the detached fleshy onion scale pieces, whereas the negative RSN test was determined by the lack of clearing pigment on at least 2 of the detached fleshy scale pieces. The RSN assay experiments were repeated.

The RSN assay was completed at WSU similarly, with minor modifications. outer dry scales were removed, and the bulbs were wiped with a paper towel moistened with 70% isopropanol. The neck and basal plate from each bulb were removed using a knife wiped with 70% isopropanol, and the bulbs split longitudinally and again crosswise through the middle.. Each scale piece was surface-sterilized in 0.75% sodium hypochlorite for 1 min, rinsed twice in sterile de-ionized water, and blotted dry with sterilized paper towels. Plastic flats (27.3 cm x 53.8 cm x 6.2 cm) were lined with two layers of moistened paper towelling, and pipette tip trays were placed on the towelling. Three onion scales were placed on each tray with the convex surface facing up, and three scales were inoculated per strain or treatment, as described above, lids placed on the flats, scale pieces were incubated in the dark at 22 °C for 4 days, and then evaluated for necrosis as described above.

#### Foliar pathogenicity assay

The ability of each of the bacterial strains, 20WA0182^T^, CCUG 14506^T^, and BD 251, to cause symptoms on onion foliage was assessed at the University of Pretoria using 18-week-old onion plants of the cv. Ranchero. The tip of each of the six replicates of onion leaves per plant was cut using scissors and sterilized with 70% ethanol for each strain. A 20-μl droplet of inoculum of the relevant bacterial suspension was placed on the cut surface of each leaf of each plant. Six onion plants were treated similarly with 1X PBS a negative treatment. The onion plants were placed in a greenhouse set at 25 ± 3 °C with 12 h/12 h of light/ dark per day. The plants were evaluated daily for seven days for symptom development. A bacterial strain was considered pathogenic when a necrotic lesion developed at the site of inoculation and extended at least 5 cm from the cut edge down the leaf, with three or more of the six inoculated leaves displaying lesions per plant. A bacterial strain was deemed negative for foliar pathogenicity in the absence of necrosis or if less than three of the inoculated leaves on a plant developed lesions at least 0.5 cm long.

Foliar pathogenicity was tested similarly at WSU using seedlings of the cv. Ranchero at approximately the five-true-leaf growth stage. Three plants growing in each 12.5-cm-diameter pot were inoculated for each strain or treatment. Two leaves per plant were cut at 5 cm below the leaf tip using scissors that had been surface-sterilized with 70% isopropanol and dried. A 20 μl droplet of inoculum was placed on the cut surface of the two leaves of each of the three plants, for a total of six leaves inoculated per strain. Inoculated plants were maintained in the greenhouse at 25–28 °C, with 12 h/12 h of light/ dark per day and evaluated seven-days post-inoculation. A strain was considered pathogenic to foliage if at least four of the six inoculated leaves developed a necrotic lesion extending down the leaf at 0.5 cm. A strain was negative for foliar pathogenicity if the lesions were 0.5 cm long on at least 4 of the 6 inoculated leaves. Foliar inoculations were repeated if only three of the six inoculated leaves had lesions at 0.5 cm long.

## Phylogenetic analyses based on 16S rRNA gene and housekeeping gene sequences

Genomic DNA of strain 20WA0182^T^ was extracted using the Zymo Quick-DNA Miniprep kit (Zymo Research, California, USA). To confirm the genus identity of strain 20WA0182^T^, the extracted DNA was amplified using the polymerase chain reaction (PCR) assay based on primers targeting the 16S rRNA gene. The 16S rRNA primer sequences for a forward reaction is 27 (5’- AGA GTT TGA TCC TGG CTC AG -3’) and for a reverse reaction 1492 (5’- CGG TTA CCT TGT TAC GAC TT-3’) (24). The PCR reaction was carried out in a total volume of 25 μl per tube by incorporating a 2.5 μl of 10X buffer that contained 15 mM MgCl_2_, 2.5 μl of 2 mM dNTPs, 1 μl of 25 nmoles of each of the 27F and 1492R primers, 0.15 μl of 5 U/μl DreamTaq DNA polymerase (Thermo Scientific, Pennsylvania, USA), 17.85 μl of nuclease-free water, and 1 μl of template DNA. Amplification PCR was carried out using a BioRad thermocycler (BioRad, California, USA) with temperature conditions for initial denaturation at 96 °C for 3 mins; then 25 cycles of 96 °C for 30 s, annealing at 55 °C for 30 seconds, and extension at 72 °C for 1 min; with a final extension at 72 °C for 5 mins. The amplified DNA was visualized on a 1% agarose gel stained with GelRed Nucleic Acid Gel Stain (Biotium, California, USA) using a BioRad Transilluminator. The amplified DNA was purified using the GeneJet PCR purification kit (Thermo Scientific, Pennsylvania, USA) following the manufacturer’s protocol. Sanger sequencing was conducted at the University of Pretoria’s sequencing facility. The sequencing PCR reaction mixture was done by mixing 1 μl of the purified PCR amplicon (Mushtaq et al. 2023), 5 μl of 5X sequencing buffer, 0.5 μl of BigDye Terminator (Thermo Scientific, Pennsylvania, USA) 0.5 μl of 25 nmoles forward primer, and 6.5 μl of nuclease-free water. The sequencing PCR cycling conditions were 96 °C for an initial denaturation for 30 s; followed by 25 cycles of 96 °C denaturation for 10 s, annealing at 55 °C for 5 s, and elongation at 60 °C for 4 mins; and held at 12 °C for 5 mins (Brady et al. 2013). The resulting sequencing mixture was purified using an ethanol precipitation method, a 12 μl aliquot of the sequencing PCR reaction was mixed with 2 μl of 3M sodium acetate, 42 ul of 99% ethanol alcohol, and 112 μl nuclease-free water. The mixture was incubated on ice for 30 min and centrifuged at 10,000 ×g for 30 min. The supernatant was discarded, and the DNA pellet washed with 250 μl of 70% ethanol, followed by centrifugation at 10,000 ×g for 10 min. The wash step was repeated, and the DNA pellet was air-dried, and Sanger sequenced.

The sequences were aligned with the reference 16S rRNA sequence of *Ewingella* strains using ClustalW (Thompson et al. 2003) and trimmed manually to create a uniform sequence alignment using BioEdit 7.0 (Hall et al. 2011). The 16S rRNA gene sequence of strain 20WA0182^T^ was deposited in GenBank with the Accession Number OR790537 and SRA Accession PRJNA1035941. For tree construction, Kimura 2 parameters were chosen through the Akaike Information Criterion (AIC) using PhyML 3.0. (Lefort et al. 2017) with 1000 bootstrap support and visualised on MEGA X (Kumar et al. 2018).

To identify strain 20WA0182^T^ at the species level, multilocus sequence analysis (MLSA) approach based 4 housekeeping genes, *atpD* (ATP synthase β-subunit), *gyrB* (gyrase β-subunit), *infB* (translation initiation factor IF-2), and *rpoB* (RNA polymerase β-subunit), was used (Brady et al. 2022). The nucleotide sequences of the 4 housekeeping genes sequences were extracted from genomes available on the NCBI database, and a local BLASTn query was created against strain 20WA0182^T^ as well as from the genomes of reference strains (Fig. 5). Gene alignments were manually corrected using BioEdit 7.0 (Larkin et al. 2007), yielding sequence lengths of 605 nt for *atpD*, 744 nt for *gyrB*, 649 nt for *infB,* and 1,009 nt for *rpoB*. The 4 housekeeping genes were concatenated using MEGA X (Kumar et al. 2018). The Kimura 2-parameter was used to construct a maximum likelihood phylogenetic tree with 1000 bootstrap replicates (Lefort et al. 2017).

## Genomic features

Whole genomic DNA was extracted from strain 20WA0182^T^ using the Qiagen Puregene Yeast/Bacteria Kit B (Qiagen, Maryland, USA) following the manufacturer’s protocol. Genomic libraries were prepared using the NEBNext Ultra II DNA Library Prep Kit for Illumina (New England Biolabs, Massachusetts, USA). The sequencing was done using the Illumina MiSeq platform with a 2 x 150 nt dual indexing (Inqaba Biotec, Pretoria, South Africa). The raw reads were quality checked using FastQC 0.12 (Andrews 2010), followed by adapter trimming and removal of low-quality reads using Trimmomatic 0.39 (Bolger et al. 2014). The resulting reads were assembled *de novo* using SPAdes 3.15 (Bankevich et al. 2012) BUSCO 5.7 (Seppey et al. 2019) was used to generate a completeness report of the assembled genome of strain 20WA0182^T^.

The genome .gff file from Prokka 1.13 (Seemann 2014) was used to generate core alignment between species of *Ewingella* using Roary 3.11 (Page et al. 2015). An alignment based on 3653 core genes was used to construct a core phylogenetic tree using RAxML 3.0 (Stamatakis 2014) and general time reversible (GTR) (Lefort et al. 2017; Stamatakis 2014; Zwickl and Holder 2004), and the tree was visualized on MEGA X (Kumar et al. 2018).The average nucleotide identity (ANI) analysis was determined for strain 20WA0182^T^ with *Ewingella* strains. ANI calculations were executed using the JSpecies platform with the BLAST algorithm accessible at https://jspecies.ribohost.com/jspeciesws/ (Richter et al. 2016). To enhance precision, the Usearch algorithm, available at https://www.ezbiocloud.net/tools/ani (Yoon et al. 2017) was also used. Digital DNA-DNA Hybridization (dDDH) was calculated using the genome-to-genome distance calculator 3.0 (GGDC) with formula 4 as the deciding criterion (https://ggdc.dsmz.de/ggdc.php) (Meier-Kolthoff et al. 2022).

## Morphology and physiology

Determination of the cell size and morphology of strain 20WA0182^T^ was conducted using scanning electron microscopy (FEGSEM: Zeiss 540 Ultra) at the Laboratory for Microscopy and Microanalysis, University of Pretoria. The strain 20WA0182^T^ was grown on NB over night in a shaker incubator at 170 rpm. After incubation, it was treated with 2.5% formaldehyde, and fixed onto a microscope grid for 16 h, followed by a wash in 1X PBS. Subsequently, a 1% osmium tetroxide solution was applied to the bacterial suspension for fixation. The fixed suspension was washed with 1X PBS, and it was dehydrated using 30%, 50%, 70%, 90%, and 100% ethanol for 15 mins for each wash. Ethanol was washed from the grid with propylene oxide, and 100% resin was added to facilitate observation of the bacterial cells using a transmission electron microscope (FEGTEM:Jeol 2100) at the Electron Microscope Unit, Veterinary Campus, University of Pretoria. Motility was also observed by using the transmission electron microscope. Gram staining was done using a Gram staining kit (Sigma-Aldrich, Massachusetts, USA), and the bacterial cells were observed for the Gram stain reaction using a light microscope (Zeiss LM: Life Sciences, Jena, Germany).

The response of strain 20WA0182^T^ to saline was evaluated over intervals of 1 to 8% (w/v) sodium chloride (NaCl), incorporated into a medium containing 3 g beef extract and 5 g peptone per 1 litre of distilled water (Brady et al. 2022). The range of temperatures the bacterium could tolerate and remain viable was measured in triplicate by growing the strain on NA at 4, 10, 15, 20, 25, 28, 30, 35, 37, 41, and 43 °C for 24–48 h. To assess the range in pH tolerance, the bacterium was exposed to pH intervals of 3 up to 10 in NB and adjusted using sodium acetate and acetic acid. To determine if the bacterium is a facultative anaerobe, mineral oil (Clicks, Pretoria, South Africa) was added to the surface of a bacterial suspension in NB and grown for 48 h. The mineral oil acted as a barrier to oxygen (Khanal et al. 2023). The catalase test was done using 3% v/v hydrogen peroxide to measure the presence or absence of the oxidative enzyme catalase. An oxidase activity test was performed using oxidase strips (Sigma-Aldrich, Massachusetts, USA) to determine the presence or absence of the cytochrome oxidase enzyme. Carbon source utilization and acid production were assessed by culturing strain 20WA0182^T^ overnight as well as the type strain of *E. americana*, CCUG 14506^T^, at 28 °C on BUG agar (Biolog, California USA) Gen III microplates and on NA for API 20NE strips (bioMérieux, Craponne, France), following the manufacturer’s protocol.

## Results and discussion

### Pathogenicity tests

**Fig. 2:**
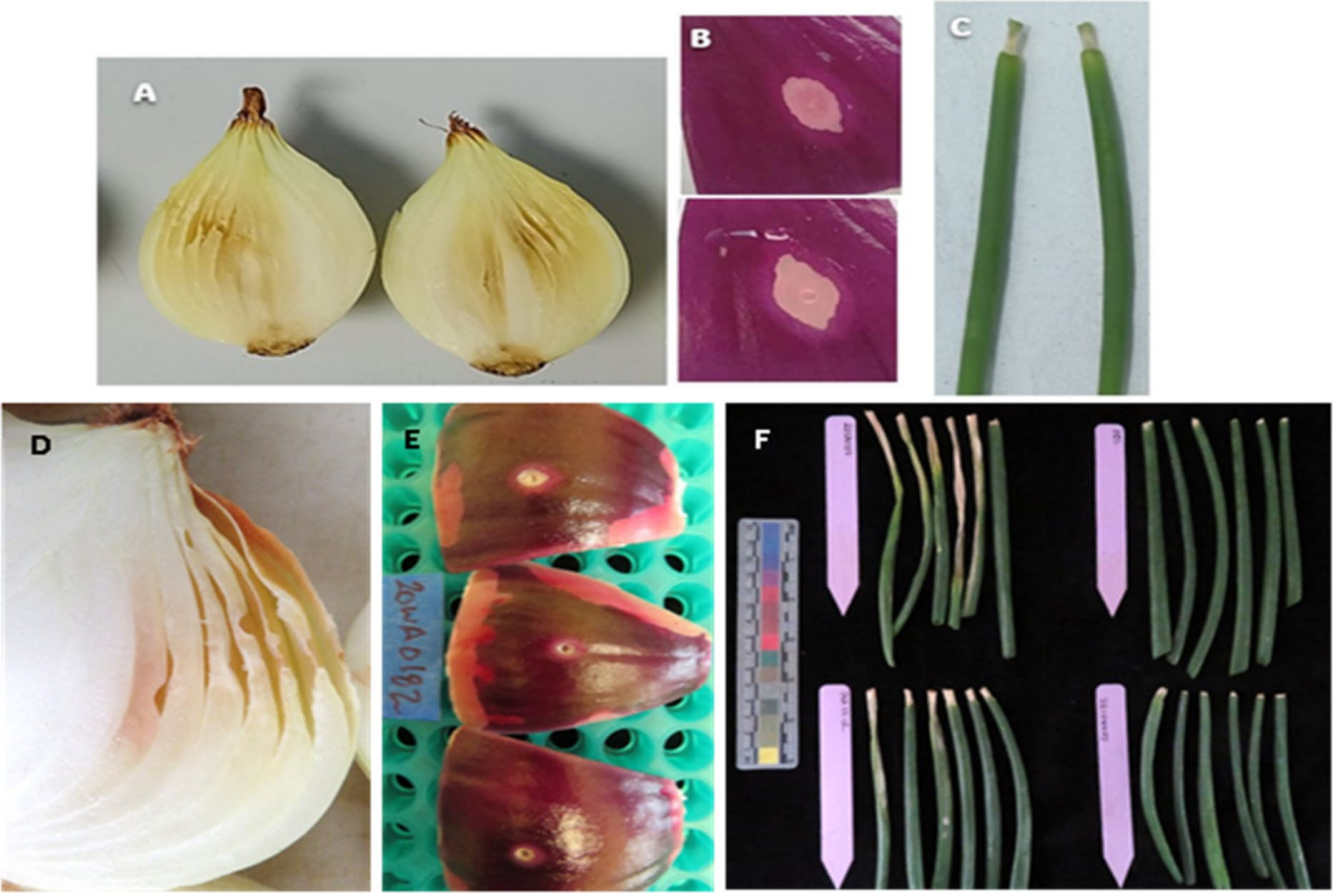
Pathogenicity of bacterial strain 20WA0182^T^ of *Ewingella* isolated from a symptomatic onion plant in Washington State, and tested for pathogenicity to onion at the University of Pretoria (**A**–**C**) and Washington State University (WSU) (**D**–**F**) using: A and D) an onion bulb assay, B and E) the red scale necrosis (RSN) assay described by Stice et al. (2021), and C and F) a foliar assay. A and D) Bulbs inoculated with 20WA0182^T^ developed discolouration and rot along the point of injection 12–14 days post-inoculation. B) In the RSN assay, 20WA0182^T^ caused a necrotic clear zone around the point of inoculation, although the zone of scale clearing was larger when tested at the University of Pretoria (B) than at WSU (E). E) Strain 20WA0182^T^ caused limited necrotic lesions at least 0.5 cm long from the point of inoculation at the cut edge of onion foliage of the cv. Ranchero, when inoculated at the University of Pretoria. However, 20WA0182^T^ did not cause necrotic lesions when inoculated onto leaves of cv. Ranchero at WSU (F, lower right leaves) compared to necrotic lesions caused by strain PNA97-1R of *Pantoea ananatis* (F, lower left leaves) and strain 20CA0107 of *Burkholderia gladioli* (F top left leaves), and leaves of the negative control plants treated with phosphate buffered saline (F, top right leaves)

For the pathogenicity assays completed at the University of Pretoria, strain 20WA0182^T^ caused mild symptoms on onion bulbs, small lesions in the RSN assay, and limited lesions on foliage compared to those of *P. ananatis* BD 251**.** Onion bulbs inoculated with strain 20WA0182^T^ showed brown discolouration and necrosis of the inner fleshy scales as well as slight shrinkage of scales around the site of injection (Fig. 2A) on three of the five replicate bulbs inoculated with strain 20WA0182^T^ in both repeats of the bulb assay. Symptoms caused by *P. ananatis* BD 251 were more severe than those caused by strain 20WA0182^T^, including maceration of the tissue. None of the bulbs injected with 1X PBS or the type strain of *E. americana*, CCUG 14506^T^, developed symptoms of bulb rot. For the bulb assay completed at WSU, the positive control strain PNA97-1R of *P. ananatis* caused brown discolouration, water-soaking, and collapse of the fleshy scales around the inoculation site, and strain 20WA0182^T^ caused a lighter brown discolouration and collapse of the fleshy scales at the site of injection, for all five inoculated bulbs for both strains (Fig. 2D). Strain Eco003 of *E. coli* and the 1X PBS buffer treatment did not cause symptoms in any of the bulbs. Symptoms observed in inoculated bulbs at WSU were similar to those observed at the University of Pretoria.

For the RSN assay, strain 20WA0182^T^ and BD 251 both caused clearing of the red pigment and necrosis of tissue around the site of inoculation on the detached scales (Fig. 2B). This was consistent for all three replicate scales in each repeat of the RSN assay, for both bacterial strains. In contrast, CCUG 14506^T^ of *E. americana* and the negative treatment with 1X PBS did not cause clearing of the red pigment, and the detached scales remained asymptomatic. The RSN assay results were consistent when repeated. For the RSN assay completed at WSU, the positive control strains PNA97-1R and Bgd015 both caused clearing of the red pigment on the scale, extending out from the wound site, for all three scales inoculated per strain. A small, dry, sunken lesion surrounded by a light halo developed on the scales inoculated with strain 20WA0182^T^ (Fig. 2E). A lesion did not develop at the wound site of scales inoculated with Eco003 or 1X PBS buffer. The WSU RSN assay results differed from the RSN assay results at the University of Pretoria, where the extent of red scale-clearing caused by strain 20WA0182^T^ was similar to that caused by the positive control strains PNA97-1R and Bgd015.

For the onion foliar pathogenicity assay completed at the University of Pretoria, light brown discolouration was observed within 3 days of inoculation, followed by necrosis and desiccation of the tissue beneath the cut surface of three or more inoculated leaves per replicate, with lesions 0.5 cm long for both strain 20WA0182^T^ and BD 251 (Fig. 2C). The type strain *E. americana*, CCUG 14506^T^, caused very small necrosis of inoculated leaves that did not differ from symptoms on leaves treated with PBS. At WSU, onion leaves inoculated with the positive control strain PNA97-1R each developed a necrotic lesion that extended 0.5 cm or more down the leaf by seven days after inoculation (Fig. 2F, lower left leaves). Necrotic lesions of this length were not observed on plants inoculated with strain 20WA0182^T^ (Fig. 2F, lower right leaves) or 1X PBS (Fig. 2F, upper right leaves). Results were consistent when the foliar assay was repeated at WSU. The lack of foliar pathogenicity of strain 20WA0182^T^ on the cv. Ranchero in the WSU assays differed from the mild lesions observed with this strain inoculated onto leaves of the same cultivar at the University of Pretoria.

**Fig. 3:**
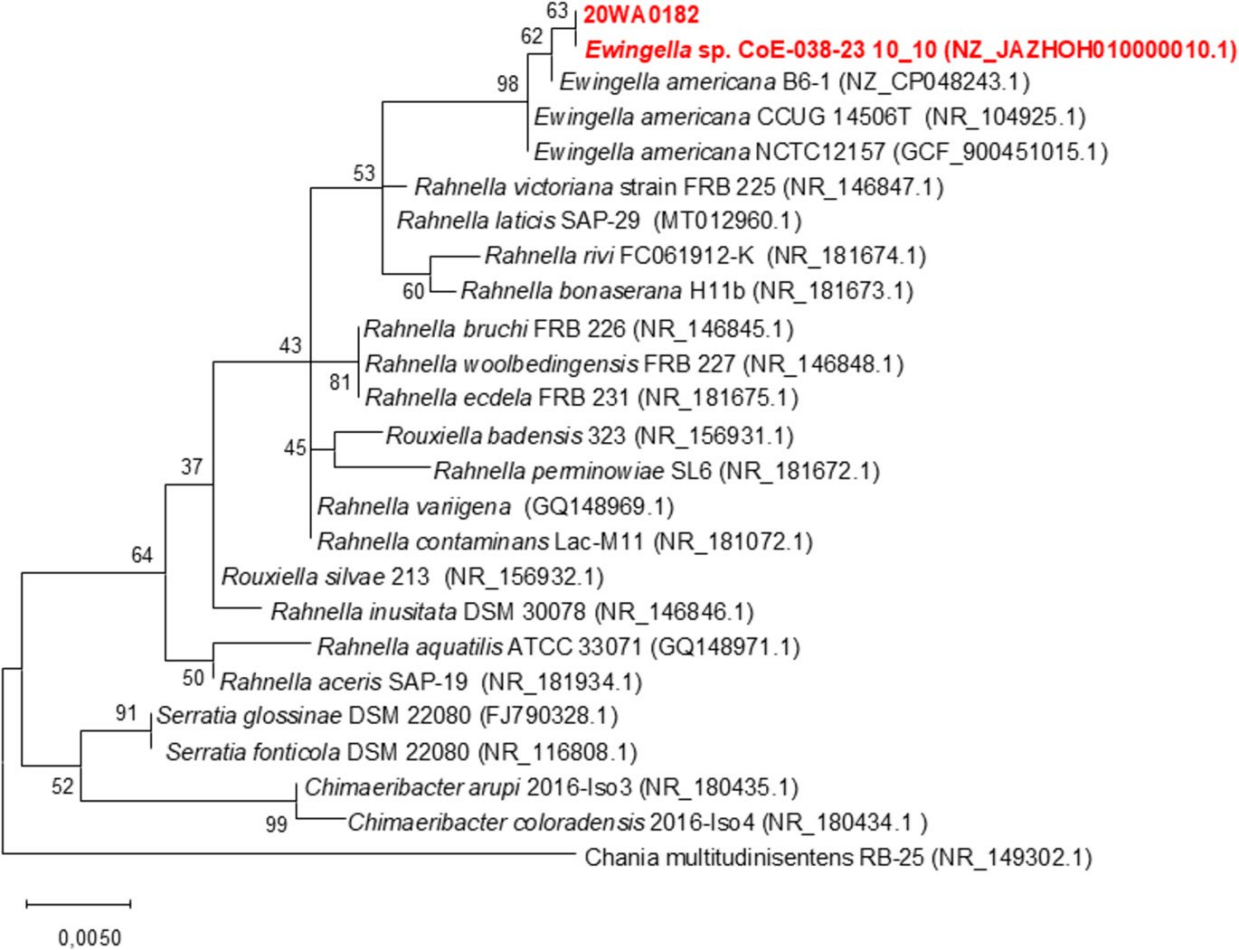
A maximum likelihood phylogenetic tree of sequences of the 16S rRNA gene for classification of bacterial strain 20WA0182^T^ isolated from the neck of a symptomatic onion plant in Washington State, USA compared to other strains in *Yersiniaceae*. A Kimura 2-parameter nucleotide substitution model was used to construct the phylogenetic tree. Strain 20WA0182^T^ clustered closely with *E*. *americana*, but on a separate branch. The scale bar represents the evolutionary distance between nodes

Re-isolations were performed from symptomatic bulbs, RSN scales, and inoculated foliage. Bacterial colonies that exhibited morphology consistent with the original strain 20WA0182^T^ were examined further to fulfil Koch’s postulates. DNA was extracted from these re-isolates and strain 20WA0182^T^ using the Quick-DNA Miniprep kit (Zymo Research, California, USA), following the manufacturer’s protocol. The extracted DNA was visualized on a 1% agarose gel. The 16S rRNA gene of these reisolated strains was sequenced as detailed below in this study to confirm the identity of the reisolates from onion bulb rot (Fig. 3).

Strain 20WA0182^T^ clearly is pathogenic to onion bulbs based on positive bulb assays and positive RSN assay results at both locations of testing. The strain was originally isolated from onion neck tissue at the lower end of a necrotic lesion that developed at the point of attachment of the fourth-oldest leaf to the neck. The strain did not cause symptoms in the foliar pathogenicity assay completed at WSU and was weakly pathogenic in the foliar assay with the same cultivar at the University of Pretoria. Cells of strain 20WA0182^T^ likely were splash-dispersed from the soil into the axil of the leaf at the point of attachment to the neck in the centre-pivot irrigated onion crop in the semi-arid Columbia Basin. The hot summer temperatures combined with centre-pivot irrigation may have created ideal conditions for the establishment of 20WA0182^T^ in the axil of the leaf, resulting in the initiation of the necrotic lesion that extended into the protected tissue in the neck.

### Phylogenetic analyses based on 16S rRNA gene and housekeeping gene sequences

**Fig. 4:**
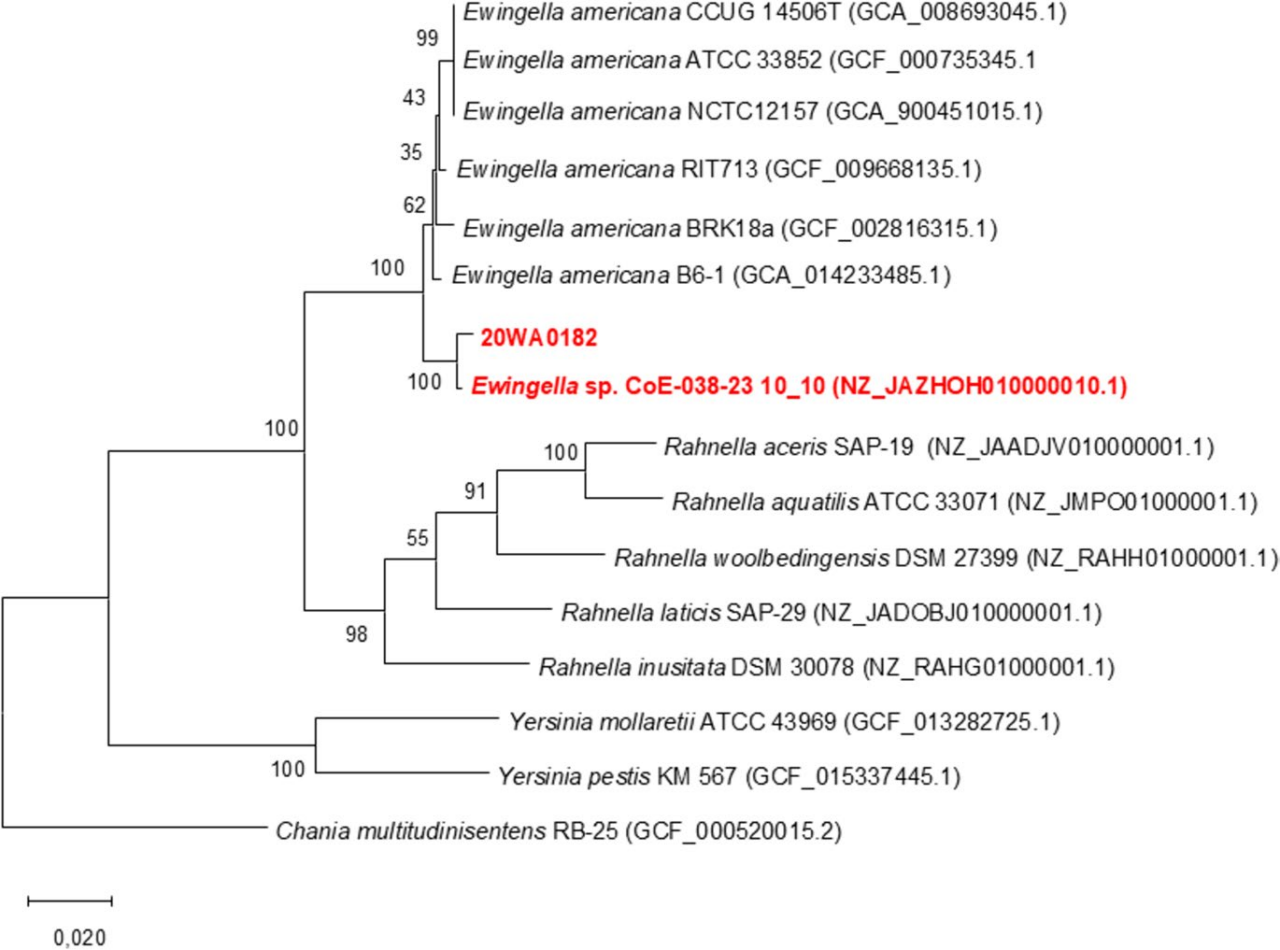
Multilocus sequence analysis (MLSA) based Maximum Likelihood phylogenetic tree for the four housekeeping genes (*atpD, gyrB, infB*, and *rpoB*), constructed using the Kimura 2-parameter model with a discrete Gamma distribution (Kimura 1980), to determine the relationship and genus identification of strain 20WA0182^T^ obtained from the neck of a symptomatic onion plant in Washington State, USA. with related strains in *Yersiniaceae* The tree shows a separate node for 20WA0182^T^ within the genus *Ewingella*

The 16S rRNA sequence of strain 20WA0182^T^ consisted of 1,331 nt. Strain 20WA0182^T^ shared a 16S rRNA sequence similarity with strain *Ewingella* sp. CoE-038-23 isolated from artisanal Montenegrin komanski cheese and with that of *E. americana* B6-1, which was isolated from the mushroom *Flammulina filiformis* (Liu et al. 2020) forming a branch with 98% bootstrap support with other *Ewingella* strains (Fig. 3). Although strain 20WA0182^T^ clustered closely with *E. americana*, it formed a distinct node alongside other genera in *Yersiniaceae*, an indication of strain 20WA0182^T^ as a novel species under the genus *Ewingella.* The MLSA phylogenetic tree revealed that strain 20WA0182^T^ formed a distinct, single branch separate from the other bacterial strains evaluated (Fig. 4). Strong bootstrap support (100%) indicated that 20WA0182^T^ belongs to the genus *Ewingella*. However, the elongated branch of strain 20WA0182^T^ separated this strain away from the *E. americana* strains (Fig. 4). This supports the classification of strain 20WA0182^T^ as a novel species of *Ewingella*.

### Genomic features

The draft genome of strain 20WA0182^T^ was deposited in GenBank and assigned Accession Number JAWUDN000000000, Completeness of the genome is 99.2%. The draft genome of strain 20WA0182^T^ consisted of 4,604,541 nt distributed across 25 contigs, with the largest contig containing 1,410,959 nt, an N50 value of 382,860 nt, and a DNA G+C content of 53.8 %. The genome consists of 4,271 genes, where 4,204 genes were for coding DNA sequences (CDS), consisting of 1 rRNA gene, 4 tRNA, and 1 tmRNA. The core phylogenetic tree distinctly separated strain 20WA0182^T^ from *E. americana* strains, as strain 20WA0182^T^ formed a distinct branch alongside *Ewingella* sp. CoE-038-23 suggesting they belong to the same novel species (Fig. 4). This further supports the designation of strain 20WA0182^T^ as a novel species of *Ewingella.*

**Fig. 5:**
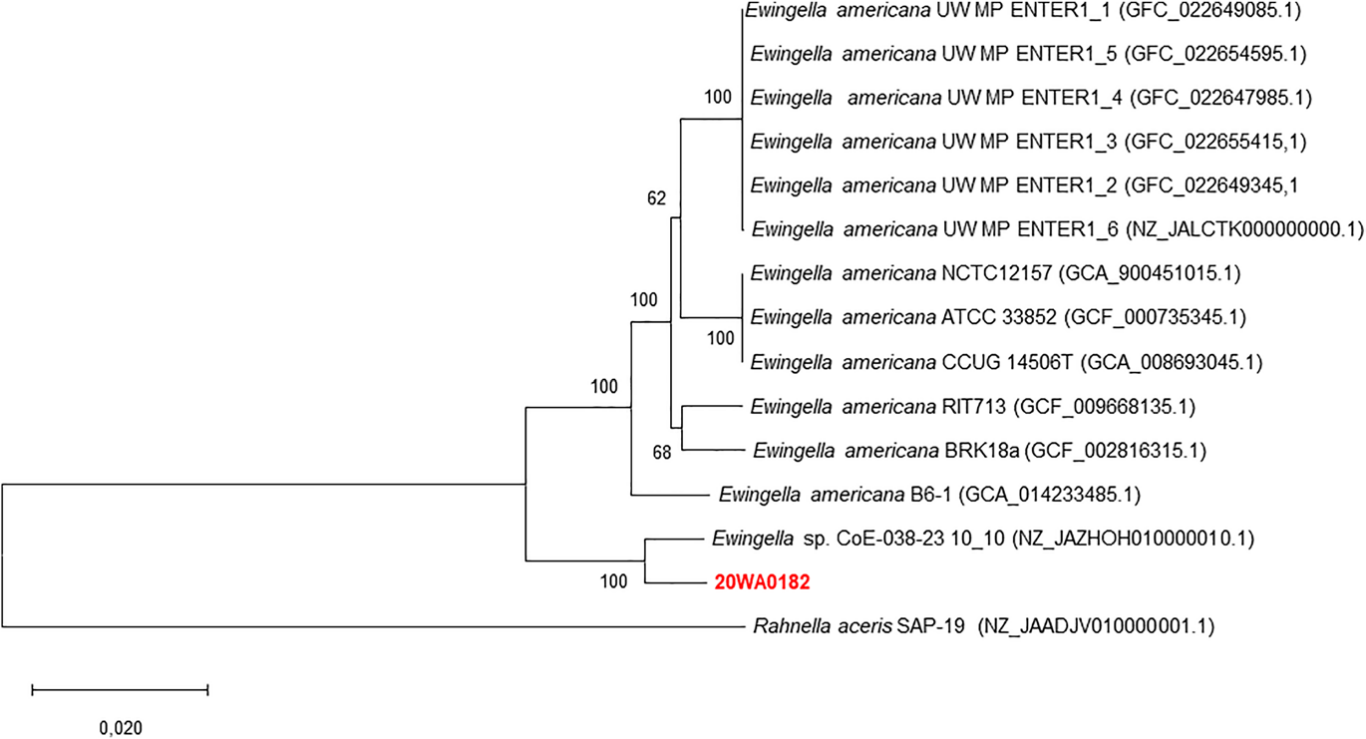
A core phylogenetic tree constructed using RAxML 3.0 (Stamatakis 2014) based on 3653 core genes from strain 20WA0182^T^ obtained from a symptomatic onion plant in Washington State, USA, with genomes of *E. americana* strains. The phylogenetic tree was visualised using MEGA X (Kumar et al. 2018; Page et al. 2015)

The ANI results are presented in Table S1, with results below the cut-off of 95% for being the same species. Strain 20WA0182^T^ shared great similarities of above 95% with two undescribed species *Ewingella* sp. CoE-038-23 and *Ewingella* sp. 33_S47 showing they may be from the same proposed species. The ANI scores observed ranged from 92.85 to 93.96%. These values fell slightly below the established cut-off of 95% needed for classifying strains as the same species of prokaryotes as *E. americana* strains. Strain *Ewingella* sp. 33_S47 was not used for further analysis due to its incompleteness. Furthermore, digital DNA–DNA hybridization (dDDH) scores ranged from 56.0 to 56.2%, which is less than the prescribed 70% cut-off for a strain to be identified as a prokaryotic species, providing further evidence of strain 20WA0182^T^ and *Ewingella* sp. CoE-038-23 to be classified as a novel species of *Ewingella* as they share a dDDH of 81.0, as represented in Table S2 (Fig. 5).

### Morphology and physiology

The size of strain 20WA0182^T^ cells was 0.74 µm x 1.02 µm, and the cells were rod-shaped (Fig. S1a). bacilli, Gram-negative, and facultative anaerobe. The strain 20WA0182^T^ was motile and possessed polar flagella (Fig. S1b). The strain 20WA0182^T^ grew at saline concentrations of 1 to 6% (w/v) after 48 h of incubation, with an optimal saline concentration of 5%. The bacterium grew at 4 to 37 °C on NA, at an optimum temperature of 28 °C. The bacterium developed slightly elevated, creamy white, opaque colonies with smooth margins on NA. The observed characteristics were similar to the type strain *E. americana* CCUG 14506^T^. Strain 20WA0182^T^ and *E. americana* CCUG 14506^T^ were able to grow at a pH range of 6 to 9 when incubated for 24 h, with an optimum pH of 7. The bacterium was catalase-positive but negative for oxidase activity.

The biochemical characteristics of strain 20WA0182^T^ were compared with those of *E. americana* CCUG 14506^T^ in Table 1. Biolog results for strain 20WA0182^T^ and *E. americana* CCUG 14506^T^ showed both strains can utilize a range of carbohydrates and amino acids, such as D-trehalose, gentiobiose, stachyose, N-acetyl-D-glucosamine, N-acetyl-D-galactosamine, D-glucose-6-PO4, fructose, L-alanine, L-glutamic acid, L-histidine, D-galacturonic acid, L-galactonic acid, D-gluconic acid, and D-glucuronic acid. However, strain 20WA0182^T^ showed to have its own unique metabolic traits that distinguish it from *E. americana* CCUG 14506^T^, including the utilisation of D-maltose, D-cellobiose, sucrose, D-turanose, glycerol, β-methyl-D-glucoside, L-aspartic acid and L-serine where *E. americana* CCUG 14506^T^ was not able to metabolize these compounds. In the API 20NE tests, 20WA0182^T^ and *E. americana* CCUG 14506^T^ shared positive reaction results for several substrates, such as 2-nitrophenyl ß D-galactopyranoside, L-lysine, L-ornithine, urea, Voges-Proskauer, D-glucose, and D-salicin. Interestingly, strain 20WA0182^T^ showed a positive reaction for hydrolyzing gelatin and reducing potassium nitrate, while *E. americana* CCUG 14506^T^ did not. Strain 20WA0182^T^ reacted negatively to D-mannitol, myo-inositol, and D-melibiose, consistent with *E. americana* CCUG 14506^T^. The distinct biochemical characteristics observed in strain 20WA0182^T^ compared to *E. americana* CCUG 14506^T^ offer compelling evidence for considering the unique classification of 20WA0182^T^ as a potential novel species within the genus *Ewingella*.

**Table 1:** Biochemical reaction tests done for strain 20WA0182^T^ isolated from a symptomatic onion plant in Washington State, USA, compared with *E. americana* CCUG 14506^ T^

Test	20WA0182^T^	*E. americana* CCUG 14506^ T^
Catalase	+	+
Oxidase	–	*–*
*Biolog Gen III:*		
D-Maltose	+	^–^
D-Trehalose	+	+
D-Cellobiose	+	–
Gentiobiose	+	+
Sucrose	+	–
D-Turanose	–	+
Stachyose	–	–
β-Methyl-D-Glucoside	+	–
D-Raffinose	–	–
N-Acetyl-D-Glucosamine	+	+
N-Acetyl-D-Galactosamine	+	+
Glycerol	+	^–^
D-Glucose-6-PO4	+	+
Fructose	+	+
L-Alanine	+	+
L-Aspartic Acid	+	–
L-Glutamic Acid	+	+
L-Histidine	+	+
L-Pyroglutamic Acid	–	–
L-Serine	+	–
D-Galacturonic Acid	+	+
L-Galactonic Acid	+	+
D-Gluconic Acid	+	+
D-Glucuronic Acid	+	+
Tween 40	+	+
*API 20NE*		
2-nitrophenyl ß Dgalactopyranoside	+	+
L-arginine	–	+
L-lysine	+	+
L-ornithine	+	+
urea	+	+
Acetoin production (Voges Proskauer)	+	+
Gelatin (bovine origin)	+	–
D-glucose	+	+
D-mannitol	–	–
Myo-inositol	–	–
D-sorbitol	+	–
L-rhamnose	+	–
D-melibiose	–	–
Potassium nitrate	+	–
D-salicin	+	+

+ , -, are positive and negative reactions

### Description of *Ewingella allii* sp. nov

*Ewingella allii* (al′li.i. L. gen. n allii pertaining to *Allium cepa*, the onion host from which the type species was isolated)

The bacterial cells of *Ewingella allii* 20WA0182^T^ are Gram-negative, facultatively anaerobic rods, motile with polar flagella. The cell was 0.74 µm x 1.02 µmin when examined with scanning electron microscopy. It formed creamy white to opaque colonies with slightly elevated, smooth, circular margins on NA. Catalase positive and negative for oxidase activity. The optimal temperature for growth is 28 °C, with growth possible at 4 to 37 °C. The optimum saline concentration for growth is 5% (w/v), with growth possible at 1 to 6% saline. The bacterium grows best at a pH of 7 but can grow at a pH of 6 to 9. Pathogenicity assays provided evidence that *Ewingella allii* ^T^ is pathogenic to onion bulbs and in detached fleshy bulb scales but is non-pathogenic or weakly pathogenic on onion leaves and foliage. Strain 20WA0182^T^ tested positive for the synthesis of D-trehalose, gentiobiose, stachyose, N-acetyl-D-glucosamine, N-acetyl-D-galactosamine, D-glucose-6-PO4, fructose, L-alanine, L-glutamic acid, L-histidine, D-galacturonic acid, L-galactonic acid, D-gluconic acid, D-glucuronic acid, 2-nitrophenyl ß D-galactopyranoside, L-lysine, L-ornithine, urea, Voges-Proskauer, D-glucose, and D-salicin (Biolog gen III and API 20NE assays). The bacteria’s unique biochemical characteristics from the type strain *E. americana* CCUG 14506^T^ by the utilisation of D-maltose, D-cellobiose, sucrose, D-turanose, glycerol, β-methyl-D-glucoside, L-aspartic acid and L-serine, gelatin and reducing potassium nitrate. The proposed strain 20WA0182^T^ could not utilise D-mannitol, myo-inositol, and D-melibiose.

The type strain 20WA0182^T^ (= BD 3290^T^ = LMG 33618^T^) was isolated from a diseased onion plant showing symptoms of bacterial rot in the neck, in an onion crop in central Washington State, USA, in 2020. The genomic DNA G+C content is 53.8%.

Strain 20WA0182^T^ is deposited in the Belgian Coordinated Collections of Microorganisms/Laboratorium voor Microbiologie Gent (BCCM/LMG) = LMG 33618^T^. and the South African Agricultural Research Council (ARC) culture collection=. BD 3290^T^. The draft genome of strain 20WA0182^T^ was assigned GenBank Accession Number =JAWUDN000000000.

## Supplementary Information

Below is the link to the electronic supplementary material.

Supplementary file1 (TIF 703 KB)

Supplementary file2 (DOCX 196 KB)

Supplementary file3 (DOCX 16 KB)

## Data Availability

Strain 20WA0182T was deposited in the Belgian Coordinated Collections of Microorganisms/Laboratorium voor Microbiologie Gent (BCCM/LMG) = LMG 33618 T. and the South African Agricultural Research Council (ARC) culture collection = . BD 3290 T. The draft genome of strain 20WA0182T was assigned GenBank Accession Number = JAWUDN000000000.
