# Supplementary figures and images for: Ewingella allii sp. nov. isolated from a diseased onion plant in the Columbia Basin of Washington State, USA
Source: Antonie Van Leeuwenhoek. 2025 Jul 16;118(8):115. doi: 10.1007/s10482-025-02116-6 (PMC12267313; doi:10.1007/s10482-025-02116-6)

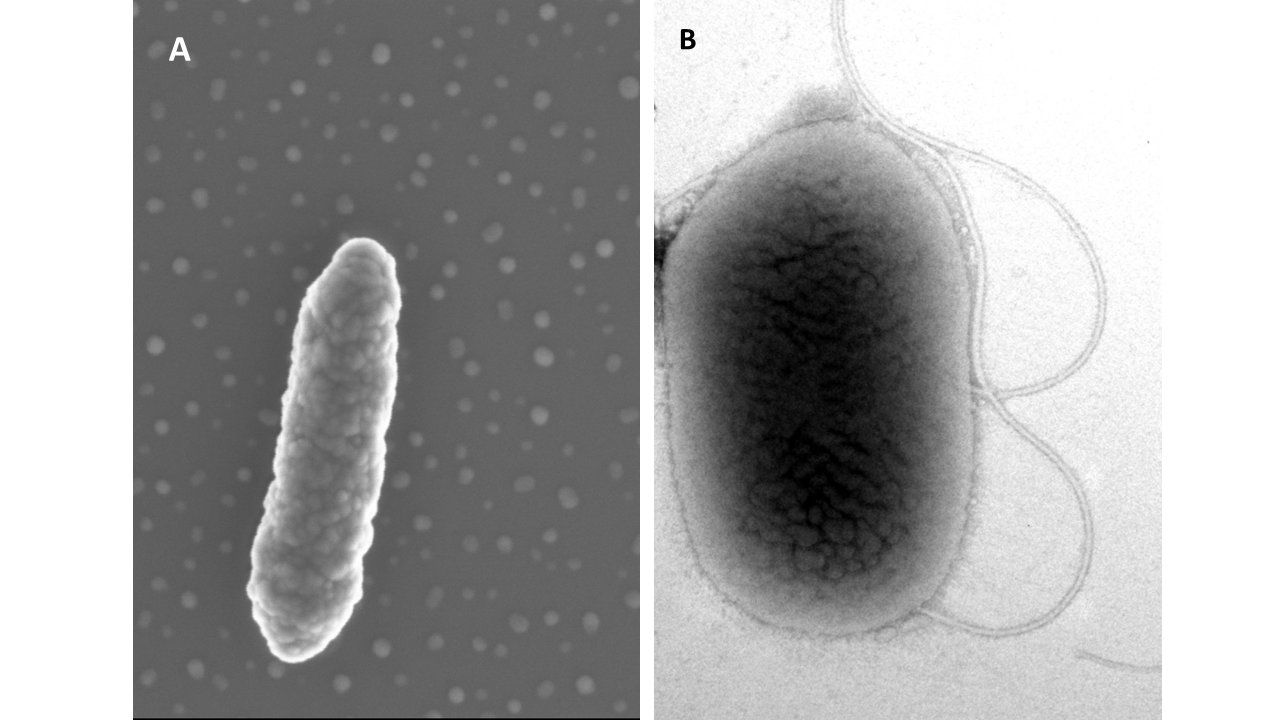

Supplement: Supplementary file 1 — Supplementary file1 (TIF 703 KB) [file 10482_2025_2116_MOESM1_ESM.tif]
