# Supplementary material for: Ewingella allii sp. nov. isolated from a diseased onion plant in the Columbia Basin of Washington State, USA
Source: Antonie Van Leeuwenhoek. 2025 Jul 16;118(8):115. doi: 10.1007/s10482-025-02116-6 (PMC12267313; doi:10.1007/s10482-025-02116-6)
Supplement: Supplementary file 2 — Supplementary file2 (DOCX 196 KB) [file 10482_2025_2116_MOESM2_ESM.docx]

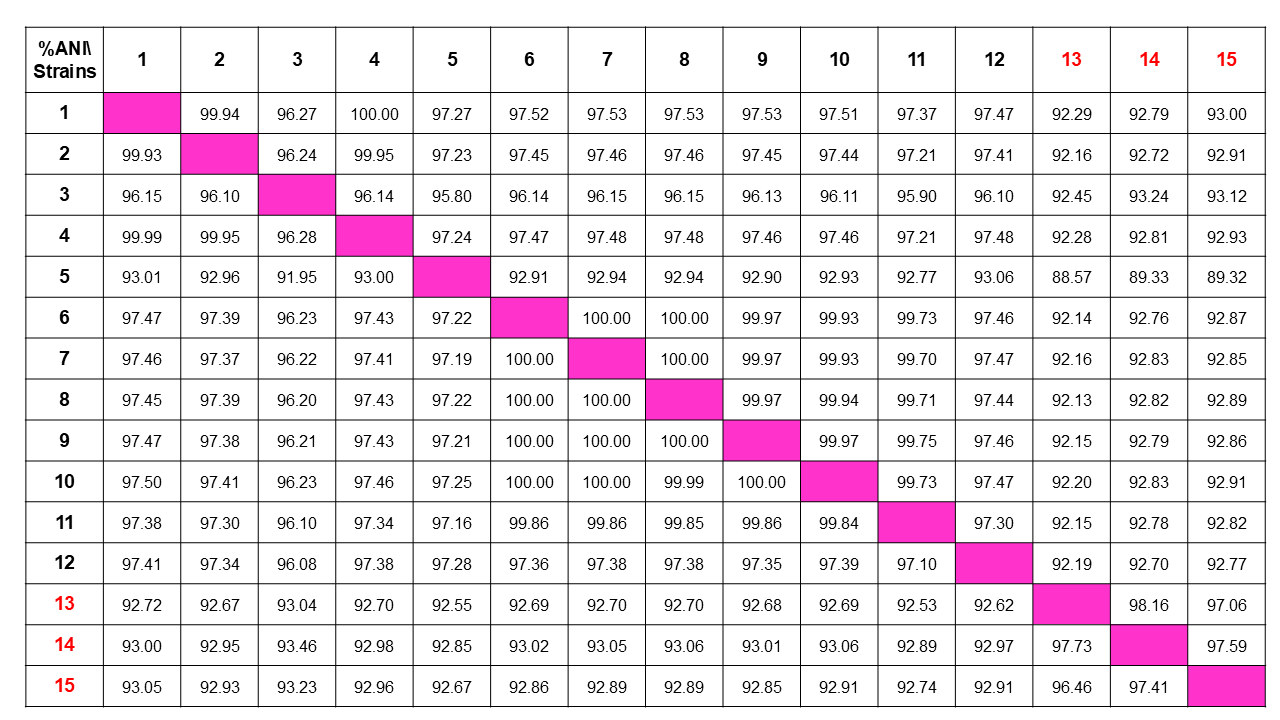
**Table S1** Average nucleotide identity (ANI) between the genome of bacterial strain 20WA0182^T^ isolated from a symptomatic onion plant in Washington State, USA and the genomes of 14 *Ewingella* strains available in GenBank. The numbers presented above the diagonal represents ANI values in (%). The numbers below the diagonal depict the alignment coverage between strains in (%).

^a^ Bacterial strains: **1**. *Ewingella americana* ATCC 33852^T^. **2**. *E. americana* NCTC 3000. **3**. *E. americana* B6-1 **4**. *E. americana* CCUG14506T. **5**. *E. americana* BRK18a. **6.** *E. americana* UW MP ENTER1_1. **7.** *E. americana* UW MP ENTER1_2. **8.** *E. americana* UW MP ENTER1_3. **9**. *E. americana* UW MP ENTER1_4. **10**. *E. americana* UW MP ENTER1_5. **11**. *E. americana* UW MP ENTER1_6. **12**. *E. americana* RIT713. **13.** *Ewingella* sp. 33_S47 **14**. Strain 20WA0182 **15.** *Ewingella* sp. CoE-038-23. Strain E4 from the NCBI database was not used as this strain was misidentified at the genus level [9].
