# Supplementary material for: Ewingella allii sp. nov. isolated from a diseased onion plant in the Columbia Basin of Washington State, USA
Source: Antonie Van Leeuwenhoek. 2025 Jul 16;118(8):115. doi: 10.1007/s10482-025-02116-6 (PMC12267313; doi:10.1007/s10482-025-02116-6)
Supplement: Supplementary file 3 — Supplementary file3 (DOCX 16 KB) [file 10482_2025_2116_MOESM3_ESM.docx]

**Table S2**: Digital DNA-DNA hybridisation values between bacterial strain 20WA0182^T^ isolated from a symptomatic onion plant in Washington State in 2020 vs. strains of *Ewingella* strains also included, a strain of *Rouxiella chamberiensis*.

| **Query** | **Genomes** | **dDDH (d4, in %)** |
| --- | --- | --- |
| 20WA0182^T^ | *Ewingella americana* ATCC 33852 | 51.2 |
| 20WA0182^T^ | *Ewingella americana* NCTC 3000 | 50.9 |
| 20WA0182^T^ | *Ewingella americana* B6-1 | 53.3 |
| 20WA0182^T^ | *Ewingella americana* CCUG 14506T | 51.0 |
| 20WA0182^T^ | *Ewingella americana* strainBRK18a | 48.8 |
| 20WA0182^T^ | *Ewingella americana*UW_MP_ENTER1_1 | 51.0 |
| 20WA0182^T^ | *Ewingella americana*UW_MP_ENTER1_2 | 51.2 |
| 20WA0182^T^ | *Ewingella americana*UW_MP_ENTER1_3 | 51.2 |
| 20WA0182^T^ | *Ewingella americana*UW_MP_ENTER1_4 | 51.1 |
| 20WA0182^T^ | *Ewingella americana*UW_MP_ENTER1_5 | 51.2 |
| 20WA0182^T^ | *Ewingella americana*UW_MP_ENTER1_6 | 51.0 |
| 20WA0182^T^ | *Ewingella* RIT713 | 51.1 |
| 20WA0182^T^ | *Ewingella* sp. CoE-038-23 | 81.0 |
| 20WA0182^T^ | *Ewingella* sp. 33_S47 | 91.4 |
| 20WA0182^T^ | *Rouxiella chamberiensis* 130333 | 21.4 |

^1^ DDH values <70% suggest lower genetic similarity and potential classification as a different species
